# Supplementary material for: Fast probabilistic whitening transformation for ultra-high dimensional genetic data
Source: PLoS Genet. 2026 Oct 28;22(10):e1012320. doi: 10.1371/journal.pgen.1012320 (PMC13734586; doi:10.1371/journal.pgen.1012320)
Supplement: Supplementary file 1 [file pgen.1012320.s001.pdf]

# Fast Probabilistic Whitening Transformation for Ultra-High Dimensional Genetic Data

Gabriel E. Hoffman[1−5*], Christian P. Dillard[1−3], Kiran Girdhar[1−3], Panos Roussos[1−5]

[1]Center for Disease Neurogenomics,
[2]Department of Psychiatry,
[3]Department of Genetics and Genomic Sciences,
Icahn School of Medicine at Mount Sinai, New York, New York, USA
[4]Center for Precision Medicine and Translational Therapeutics,
[5]Mental Illness Research, Education and Clinical Center VISN2,
James J. Peters VA Medical Center, Bronx, New York, USA.

*Contact: gabriel.hoffman@mssm.edu

## Supplementary Information

### Defining the whitening matrix

Consider data matrix $Y_{n\times p}$ covariance between columns given by $\Sigma_{p\times p}$ and each column having zero mean after centering. Defining the singular value decomposition $Y/\sqrt{n}$ as $UDV^T$, then

$$\begin{aligned} \text{cov}(Y) &= n^{-1}Y^TY && (1)\\ &= VDU^TUDV^T && (2)\\ &= VD^2V^T. && (3)\end{aligned}$$

Setting $\Sigma^{-\frac{1}{2}} = VD^{-1}V^T$, and evaluating the covariance between columns following the whitening transform gives identity covariance according to

$$\begin{aligned} \text{cov}(Y\Sigma^{-\frac{1}{2}}) &= \Sigma^{-\frac{1}{2}}\text{cov}(Y)\Sigma^{-\frac{1}{2}} && (4)\\ &= \left(VD^{-1}V^T\right)\left(VD^2V^T\right)\left(VD^{-1}V^T\right) && (5)\\ &= VD^{-1}D^2D^{-1}V^T && (6)\\ &= VV^T && (7)\\ &= I_p. && (8)\end{aligned}$$

## Evaluating whitening performance

Let $\hat{A}$ be the whitening transformation learned from the observed data so that

$$Y\hat{A} \sim \left[M\hat{A}, I_n, \hat{A}^T\Sigma\hat{A}\right]. \tag{9}$$

When $\hat{A}^T\Sigma\hat{A} = I_p$, as in Equation (**??**), this transformation produces data $Y\hat{A}$ whose columns are *exactly* uncorrelated when $\hat{A}$ is estimated from $Y$. Yet this condition can only be satisfied when the sample covariance matrix, $\hat{\Sigma}$, is invertible, and this requires $n > p$. In high dimensional data with $n < p$, so some form of regularization is needed. An exact whitening transformation no longer exists in these cases, so we want to learn $\hat{A}$ from the data so that $\hat{A}^T\Sigma\hat{A}$ is 'close' to the identity matrix by some metric. Formally, we could seek a transform so that $\|\hat{A}^T\Sigma\hat{A} - I_p\|_F$ is small, where $\|.\|_F$ is the Frobenius norm of a matrix. This is closely related to the root mean squared error of the difference between the covariance of the transformed data and the identity matrix.

The Frobenius norm of a matrix $\Omega_{p\times p}$ is defined as

$$\|\Omega\|_F = \sqrt{\sum_{i=1}^{p}\sum_{j=1}^{p} M_{i,j}^2} \tag{10}$$

so that scaling by $p$ gives

$$\|\Omega\|_F/p \;\;=\;\; \sqrt{\left(\sum_{i=1}^{p}\sum_{j=1}^{p} M_{i,j}^2\right)/p^2}, \tag{11}$$

which is the root mean squared error when $\Omega = \hat{A}^T\Sigma\hat{A} - I_p$.

## Regularizing the correlation matrix and retaining sample variances

The approach described so far shrinks all diagonal variance entries towards a single value $\nu$. While this is reasonable when the variances are all equal, most covariance matrices in practice have heterogeneous variances. Instead, we can factor out the variance terms, shrink the correlation matrix and then rescale by the variances. Let $\hat{\Sigma}_{MLE}$ have variance $\hat{\sigma}_i^2$ in the $i^{th}$ entry on the diagonal, let $Z$ be a diagonal matrix with $\hat{\sigma}_i$ in the $i^{th}$ entry and let $\hat{C}_{MLE}$ be the corresponding correlation matrix estimate so that the covariance can be decomposed according to $\hat{\Sigma}_{MLE} = Z\hat{C}_{MLE}Z$. The regularized covariance matrix retaining the sample variances along the diagonal is then

$$\hat{\Sigma}_\lambda \;\;=\;\; Z\left[(1-\lambda)\hat{C}_{MLE} + \lambda I\right]Z. \tag{12}$$

The approach described in the main text corresponds to setting $Z = I$ and estimating $\nu$ from the data. Alternatively, a second approach corresponds to estimating the standard deviation matrix, $Z$, from the data and setting $\nu = 1$. We omit the variance terms from the notation throughout for the sake of simplicity.

## Estimation of shrinkage parameter

Setting the value of the shrinkage parameter $\lambda$ is a key component for any convex combination method and many methods have been proposed under different, often nonparametric, assumptions [**? ? ? ? ? ?** ]. Following the probabilistic formulation, we take advantage of the Gaussian Inverse Wishart model and use an empirical Bayes approach to estimate the shrinkage parameter after marginalizing out the covariance. The probability of the observed data can be marginalized with respect to the unknown covariance term $\Sigma$ according to

$$p(Y|\delta,\nu) = \int p(Y|\Sigma,\delta,\nu)p(\Sigma|\delta,\nu)d\Sigma \tag{13}$$

$$= \pi^{-np/2}\frac{\Gamma_p\left(\frac{\delta+n}{2}\right)}{\Gamma_p\left(\frac{\delta}{2}\right)}\frac{|(\delta-p-1)T|^{\frac{\delta}{2}}}{|(\delta-p-1)T+S|^{\frac{\delta+n}{2}}} \tag{14}$$

$$= \pi^{-np/2}\frac{\Gamma_p\left(\frac{\delta+n}{2}\right)}{\Gamma_p\left(\frac{\delta}{2}\right)}\frac{|(\delta-p-1)\nu I|^{\frac{\delta}{2}}}{|(\delta-p-1)\nu I+S|^{\frac{\delta+n}{2}}}. \tag{15}$$

where $S = YY^T$, and we focus on the case where the target matrix is a scaled identity matrix so that $T = \nu I$.

The empirical Bayes estimates of $\delta$ and $\nu$ can be obtained from the observed data according to

$$\hat{\delta},\hat{\nu} = \underset{\delta,\nu}{\text{argmax}}\ \ell(\delta,\nu|Y). \tag{16}$$

where

$$\ell(\delta,\nu|Y) = \log p(Y|\delta,\nu) \tag{17}$$

$$= -\frac{np}{2}\log\pi + \log\Gamma_p\left(\frac{\delta+n}{2}\right) - \log\Gamma_p\left(\frac{\delta}{2}\right) \tag{18}$$

$$+\frac{\delta p}{2}\log\left(\nu(\delta-p-1)\right) \tag{19}$$

$$-\frac{\delta+n}{2}\sum_i \log\left(\nu(\delta-p-1)+d_i^2\right) \tag{20}$$

where $d_i^2$ are the eigen values of $S$. Since $\hat{\nu}$ can be estimated in closed form from the data as the mean diagonals of $S$, and $\hat{\delta}$ can be obtained subsequently using a line search, $\hat{\lambda}$ can then be obtained from $\hat{\delta}$. Importantly, this function has one parameter and the data enters only through the eigen-values $d^2$ and the prior scale, $\nu$.

### Low rank approximation

With the full spectral decomposition, the estimate of the correlation matrix

$$\hat{\Sigma}_\lambda = (1-\lambda)\hat{\Sigma}_{MLE} + \lambda\nu I \tag{21}$$

has diagonal values equal to 1. But when the data is approximated by a truncated singular value decomposition, $\hat{\Sigma}_\lambda$ no longer has diagonals of value 1 when simply setting $\nu = 1$ . Instead, we set the value of $\nu$ so that the mean of the diagonals of $\hat{\Sigma}_\lambda$ is closest to 1.

Let the target sum of the diagonal values of $\hat{\Sigma}_\lambda$ be $p$, and let

$$a = \sum_i \left[U_{1:k}\text{diag}(d^2_{1:k})U^T_{1:k}\right]_{i,i} \tag{22}$$

be the sum of the diagonals of the low rank approximation to the correlation matrix, $\hat{\Sigma}_{MLE}$. Therefore, the sum of the diagonals of $\hat{\Sigma}_\lambda$ is $(1-\lambda)a + \lambda\nu p$.

Setting this value equal to the desired sum of the diagonals, $p$, and solving for $\nu$ gives

$$\hat{\nu} = (p - (1-\lambda)a)\,/\lambda p. \tag{23}$$

This value is estimated *within* each evaluation of $\ell(.)$ so that the function has just 1 parameter.

## Properties as a function of $\lambda$

Here we can examine the behavior of the transformation as a function of $\lambda$. First, all *modified* singular values are positive, even in cases where the corresponding singular value of the observed data is zero. This is intuitive since all eigen-values of the implied covariance matrix $\hat{\Sigma}_\lambda$ must be positive for it to be invertible.

Based on results above, setting $\lambda = 0$ corresponds to the algorithmically defined whitening transformation using the sample covariance matrix where there is no influence from the prior. Indeed, Equation (**??**) shows that this corresponds to setting all singular values of the transformed data to 1, thus scaling the sample variances in all dimensions to be equal. At the other extreme, setting $\lambda = 1$ divides each singular value by $\sqrt{\nu}$ so the singular values of the transformed data are just scaled versions of the original values, and thus retains the same correlation structure. For intermediate values of $\lambda$ that are used in practice, they are shrunk using $\nu$ as a 'pivot': values greater then $\nu$ are shrunk towards the pivot and values less than $\nu$ are inflated towards the pivot.

As $\lambda \to 1$ the prior is increasingly concentrated around the target matrix and recovers the target matrix in the limit. As $\lambda \to 0$, this corresponds to an infinitely diffuse prior and recovers the sample covariance matrix in the limit. As $n$ increases for fixed $p$, a good estimator of $\lambda$ should converge to 0 so the posterior expectation is an asymptotically consistent estimator of the covariance [**?** ].

Mapping from the observed to the modified singular values depends on $\lambda$, which in turn depends on $n$ and $p$, and $\vec{d}$. Small sample sizes require stronger regularization with larger $\lambda$ values. In this case, the modified singular values retain some level of variation so the whitening transform is approximate. With increasing sample size, the effect of the prior decreases so that $\lambda \to 0$ as $n \to \infty$ with fixed $p$, so the modified singular values approach the observed singular values. At the same time, $\hat{\Sigma}_\lambda \to \hat{\Sigma}_{MLE}$

and $\text{cov}\left(Y\hat{\Sigma}_\lambda^{-\frac{1}{2}}\right) \to I$ so that the whitening transform is asymptotically exact, and approximate for finite sample size.

The out-of-sample performance of the whitening transform has received less attention. For small sample size, the whitening transform estimated from the sample covariance can give very poor out-of-sample performance and can in fact increase the correlation between features. Regularization produces better estimates of the true singular values [? ] and we demonstrate that it can also substantially improve out-of-sample whitening performance.

## Interpretation of shrinkage parameter

The sample covariance $\hat{\Sigma}_{MLE}$ requires $p(p-1)/2$ off-diagonal covariance parameters. Since the posterior expectation is a convex combination between prior and sample covariance, we can compute the effective number of parameters of the posterior estimate, $\hat{\Sigma}_\lambda$, as a function of the $\lambda$ shrinkage parameter.

Following Ye [? ], the effective number of parameters is determined by the degree to which changes in the observed data change the estimator. Letting $f_\lambda(\hat{\Sigma}_{MLE}) = (1-\lambda)\hat{\Sigma}_{MLE} + \lambda\nu I$ be the mapping from the sample covariance to the posterior covariance, Ye [? ] defines the effective number of parameters as

$$\sum_{i,j} \frac{\partial f_\lambda(\hat{\Sigma}_{MLE})}{\partial \hat{\Sigma}_{MLEi,j}}, \tag{24}$$

which is interpretable as the sum of the degree to which changes in the sample covariance change the posterior estimate. Considering only the off-diagonal covariance terms,

$$\frac{\partial f_\lambda(\hat{\Sigma}_{MLE})}{\partial \hat{\Sigma}_{i\neq j}} = 1 - \lambda, \tag{25}$$

so each covariance term uses $1-\lambda$ effective parameters. Therefore the effective number of parameters used to estimate the covariance terms is $(1-\lambda)p(p-1)/2$. Compared to the sample covariance, the effective number of parameters is thus reduced by a factor of $1-\lambda$.

As the number of features, $p$, increases with fixed sample size $n$, $\hat{\lambda} \to 1$ to apply stronger shrinkage [? ]. While the effective number of parameters for the sample covariance matrix grows quadratically with $p$, it grows slower than quadratically for the shrinkage model since $\lambda$ increases with $p$ for fixed $n$.

## Scalar summaries of the correlation or covariance matrix

The condition number of $\hat{\Sigma}_\lambda$ is defined as the ratio between the largest and smallest eigen-values:

$$\kappa_\lambda \quad = \quad \frac{(1-\lambda)d_1^2 + \lambda\nu}{(1-\lambda)d_{min}^2 + \lambda\nu}. \tag{26}$$

When the sample covariance is low rank, the smallest sample eigen-value is zero so the condition number has the simple form

$$\kappa_\lambda = \frac{(1-\lambda)}{\lambda\nu} d_1^2 + 1. \tag{27}$$

as long as $\lambda > 0$. Instead of regularizing the condition number using an ad hoc tuning parameter, or estimating it by cross validation, in the probabilistic model $\lambda$ is a function of $n$, $p$ and $\vec{d}$. As $\lambda$ increases, more weight is put on the target matrix, and the condition number approaches its minimum value of 1.

The effective variance is defined as the geometric mean of the eigen-values and satisfies a number of properties for a metric of multivariate variability [? ]. This is defined as $|\Sigma_\lambda|^{1/p}$, but can also be computed directly from the eigen-values. The effective number of features is analogous to the effective sample size based on the sum of all elements in the inverse correlation matrix [? ] which is defined as $\sum_{i,j}[\Sigma_\lambda^{-1}]_{i,j}$. The average correlation can be computed by evaluating the sum of the off-diagonal entries of the correlation matrix divided by the number of entries. The effective number of features and average correlation can be computed efficiently using the implicit correlation matrix as described below. These values can be computed for the $\Sigma_{MLE}$ matrix by setting $\lambda = 0$.

## Scaling of standard whitening transform

Constructing $\hat{\Sigma}_{MLE}$ is $\mathcal{O}(np^2)$, requires $\mathcal{O}(p^2)$ memory, and evaluating its eigen decomposition is $\mathcal{O}(p^3)$. Finally, explicitly creating the whitening matrix is $\mathcal{O}(p^2)$, and evaluating the matrix product is $\mathcal{O}(np^2)$. Estimating $\lambda$ with the probabilistic model is $\mathcal{O}(p)$, but other methods look at the entire dataset so require at least $\mathcal{O}(np)$ time. Estimating $\nu$ is negligible in comparison, and applying shrinkage to the standard whitening transformation is also negligible. This allows us to consider the standard whitening transformation as a special case of the probabilistic model with $\lambda = 0$. The time complexity of the entire procedure is therefore $\mathcal{O}(p^3 + np^2)$. The standard algorithm becomes very expensive for $p > 100$ and can be intractable for $p > 2000$ features.

## Accelerating transform using properties of linear algebra

**Identity 1.** *Let $C$ be a $p \times p$ symmetric positive semi-definite matrix, with $k \leq p$ positive eigen values. Let the eigen decoposition of $C$ be $U diag(s) U^T$ where $s_i = 0$ for $i > k$. Let the eigen-vectors be partitioned as $U = [U_1\, U_2]$, and the eigen-values be partitioned as $s = [s_1\, s_2]$ to separate the nonzero and zero components. Let raising a matrix to an exponent $\alpha$ correspond to raising the eigen-values of the matrix to $\alpha$. Then*

$$A(C + \tau I_p)^\alpha B = (AU_1)\, diag(s_1^\alpha) U_1^T B + \tau^\alpha \left(AB - (AU_1)U_1^T B\right) \tag{28}$$

*where $U_1$ and $s_1$ indicate the first $k$ eigen-vectors and values, respectively, and $A_{a\times p}$ and $B_{p\times b}$ are arbitrary matrices, $\tau$ is a positive scalar, and $I_p$ is the p-dimensional identity matrix.*

*Proof.* The derivation follows from standard properties of linear algebra, the fact that the matrix of eigen-vectors, $U$, is orthonormal, and Lemma **??** below.

$$\begin{aligned}
A(C + \tau I_p)^\alpha B &= A\left(U\text{diag}(s)U^T + \tau I_p\right)^\alpha B && (29)\\
&= A\left(U\left(\text{diag}(s) + \tau I_p\right)U^T\right)^\alpha B && (30)\\
&= AU\text{diag}(s+\tau)^\alpha U^T B. && (31)
\end{aligned}$$

While this formula removes $U$ from within the exponentiated term, it still includes $p-k$ eigen-values that are zero along with their corresponding eigen-vectors. Partitioning the non-zero and zero terms of $s$ and $U$ and into separate components produces an expression using only non-zero terms.

$$\begin{aligned}
&= A[U_1\, U_2]\begin{bmatrix} s_1 I_k + \tau & 0 \\ 0 & \tau I_{p-k}\end{bmatrix}^\alpha [U_1\, U_2]^T B && (32)\\
&= AU_1\text{diag}(s_1+\tau)^\alpha U_1^T B + AU_2(\tau^\alpha I_{p-k})U_2^T B && (33)\\
&= AU_1\text{diag}(s_1+\tau)^\alpha U_1^T B + \tau^\alpha AU_2U_2^T B && (34)\\
&= AU_1\text{diag}(s_1+\tau)^\alpha U_1^T B + \tau^\alpha A(I_p - U_1U_1^T)B && (35)\\
&= AU_1\text{diag}(s_1+\tau)^\alpha U_1^T B + \tau^\alpha(AB - (AU_1)U_1^T B) && (36)
\end{aligned}$$

□

**Corollary 1.1.** *This allows efficient evaluation of $A(C+\tau I_p)^\alpha B$ for many values of $\tau$ by precomputing $s_1$, $U_1$, $AU_1$ and $U_1^T B$.*

**Lemma 1.1.** *Since $U$ is orthonormal,*

$$\begin{aligned}
I_p &= UU^T && (37)\\
&= [U_1\, U_2][U_1\, U_2]^T && (38)\\
&= U_1U_1^T + U_2U_2^T && (39)
\end{aligned}$$

*Rearranging, it follows that $U_2U_2^T = I_p - U_1U_1^T$*

## Imputing z-statistics for tandem repeats

The VCF files from the EnsembleTR v4 [**?** ] reference panel including donors from 1000 Genomics Project was obtained from `https://github.com/gymrek-lab/EnsembleTR`. Summary statistics from a study of genetic regulation of gene expression in the human brain [**?** ] was downloaded from `https://www.synapse.org/Synapse:syn25592272`, and z-statistics from the fixed effects meta-analysis were used here.

Due to the complexity of harmonizing tandem repeats identified in sequence data, the EnsembleTR reference panel [**?** ] often has multiple alternatively alleles at a site. For example, consider the hexanucleotide repeat near C9orf72 which is encode with 25 alternative alleles (**Table S1**). When

multiple alleles are observed at a site, the alleles are modified to have multiple biallelic variants at the same site using the command: `bcftools norm -m - ${VCF}`. The z-statistics are imputed for each biallelic variant, and then combined into a single z-statistic for that site using a random effects meta-analysis that models the correlation structure between the variants [? ]. This is implemented using the `RE2C()` function in the `remaCor` package available at `https://cran.r-project.org/package=remaCor`.
