# Supplementary material for: Fast probabilistic whitening transformation for ultra-high dimensional genetic data
Source: PLoS Genet. 2026 Oct 28;22(10):e1012320. doi: 10.1371/journal.pgen.1012320 (PMC13734586; doi:10.1371/journal.pgen.1012320)
Supplement: Supplementary file 2 [file pgen.1012320.s002.pdf]

Imputed z	Alternative allele in EnsembleTR
1.46	GCCCCGCCCCGGGCCCGCCCCCGGGCCCGCCCCGACCACGCCCCGGCCCCGGCCCCGGCCCCGGCCCCGGCCCCGGCCCC
-17.16	GCCCCGCCCCGGGCCCGCCCCCGGGCCCGCCCCGACCACGCCCCGGCCCCGGCCCC
9.18	GCCCCGCCCCGGGCCCGCCCCCGGGCCCGCCCCGACCACGCCCCGGCCCCGGCCCCGGCCCCGGCCCC
20.11	GCCCCGCCCCGGGCCCGCCCCCGGGCCCGCCCCGACCACGCCCCGGCCCCGGCCCCGGCCCCGGCCCCGGCCCCGGCCCCGGCCCCGGCCC
-2.79	GCCCCGCCCCGGGCCCGCCCCCGGGCCCGCCCCGACCACGCCCCGGCCCCGGCCCCGGCCCCGGCCCCGGCCCCGGCCCCGGCCCCGGCCC
-1.29	GCCCCGCCCCGGGCCCGCCCCCGGGCCCGCCCCGACCACGCCCCGGCCCCGGCCCCGGCCCCGGCCCCGGCCCCGGCCCCGGCCCCGGCCC
8.78	GCCCCGCCCCGGGCCCGCCCCCGGGCCCGCCCCGACCACGCCCCGGCCCCGGCCCCGGCCCCGGCCCCGGCCCC

Table 1: Imputed z-statistics for tandem repeat EnsTR:chr9:27573485 associated with expression of C9orf72. Due to the complexity of harmonizing tandem repeats identified in sequence data, the EnsembleTR reference panel encodes the known hexanucleitude repeat near C9orf72 as a 62bp reference allele (GCCCCGCCCCGGGCCCGCCCCCGGGCCCGCCCCGACCACGCCCCGGCCCCGGCCCCGGCCCC) with 25 alternative alleles. The 7 most common are shown here with their imputed z-statistics.
