# Supplementary material for: Fast probabilistic whitening transformation for ultra-high dimensional genetic data
Source: PLoS Genet. 2026 Oct 28;22(10):e1012320. doi: 10.1371/journal.pgen.1012320 (PMC13734586; doi:10.1371/journal.pgen.1012320)
Supplement: Supplementary file 3 [file pgen.1012320.s009.pdf]

Response to Reviewers

We thank the Reviewers for their constructive feedback on our manuscript. Their contributions have strengthened the manuscript. We have responded to each comment below. Our response is shown in indented text, and changes to the manuscript are shown in red.

Reviewer #1:

Whitening is widely used in many analyses of genetic data. This manuscript proposes a new method for performing whitening transformations accurately and efficiently. Overall, the paper addresses an important problem, and the proposed method has the potential for broad applications in genetics and beyond. I have the following comments.

1. The abstract and introduction are more appropriate for a statistical audience. The description or scientific motivations is brief. For example, why is probabilistic whitening important for a broad audience in the field of genetics? Although the authors mentioned phenotype prediction, statistical fine-mapping, imputing z-statistics, detecting anomalous z-statistics, multiple testing correction, etc, in the introduction, they did not elaborate on the limitation of current whitening methods in the context of these analyses and how they can influence downstream findings. Discussing these motivations early in the Introduction could appeal to more researchers who may be interested in using the method. Similarly, starting the abstract with a bit more scientific motivation would help.

> We thank the Reviewer for this valuable feedback. We have updated the Abstract and Introduction:
>
> Statistical methods often make assumptions about independence between the samples or features of a dataset, yet correlation structure is ubiquitous in real data. In statistical genetics, correlation between regression summary statistics due to linkage disequilibrium (LD) between nearby variants complicates downstream analysis. Many methods use LD reference panels to account for this correlation. Whitening transformations comprise a family of methods to remove correlation structure, and are used implicitly by many common models. Here we propose a probabilistic model for data whitening, and propose parameter estimates and efficient algorithms to improve statistical and computational performance. In an application to impute z-statistics from unobserved genetic variants from a genome-wide association study of schizophrenia, the probabilistic whitening transformation, had the lowest mean square error while being up to an order of magnitude faster than other methods. Using this approach, we also identify tandem repeats that explain genetic regulatory signals for disease-relevant genes. Analyses are implemented in our novel open source R packages `decorrelate` and `imputez`.

1 Introduction

Correlation structure is ubiquitous in real world datasets. Yet statistical models often make assumptions about independence between a dataset's rows or columns that are not satisfied practice. Whitening transformations are a widely used preprocessing step to remove correlation structure in the data before performing other analyses. In general, for a data matrix $Y_{n \times p}$ with n samples and p features, the procedure computes the sample covariance matrix between features, $\Sigma_{p \times p}$, and transforms the observed data by post-multiplying by the non-unique whitening matrix $\Sigma^{-\frac{1}{2}}$ defined using standard linear algebra [1]. Transforming rows instead follows a complementary procedure.

Whitening can be used explicitly in preprocessing, but more often it is used implicitly when a covariance matrix is used internally to account for non-independence of samples or features and the whitening is implied by the linear algebra of the internal calculations. Indeed, many widely used methods such as multivariate regression, canonical correlation analysis, linear and quadratic discriminate analysis, Gaussian mixture models, Maholanobis distance, multivariate outlier detection, and linear mixed models can be reframed using an implicit whitening transformation [2–6].

The field of statistical genetics makes extensive use of implicit whitening transformations in order to account for the correlation between nearby genetic variants. This correlation structure is termed 'linkage disequlibirum' or 'LD' and is a major complication in downstream analysis of regression summary statistics (i.e. coefficient estimates or z-statistics) from genome-wide association studies [7]. It is common practice to estimate the correlation matrices from an external 'reference panel' of samples not included in the study of interest [7]. These 'out-of-sample' estimates are then used either as a preprocessing step to transform the summary statistics to be approximately uncorrelated, or internally in a statistical model. This approach has become standard in the field for phenotype prediction [8, 9], statistical fine-mapping [10–12], imputing z-statistics [13, 14], detecting anomalous z-statistics [15], multiple testing correction [16], identifying genome annotations enriched for risk variants [17], and simulating knockoff datasets with similar correlation structure [18]. While the literature on whitening transformations has focused on estimating and applying the transform on the same data [1], an understanding of the performance of these out-of-sample applications has been lacking [19].

Here, we examine the explicit whitening transformation and its properties in order to improve performance in a cases where it is used implicitly. This can improve out-of-sample performance when using modestly sized reference panels, and substantially reduce computational time.

2. The methods should be strengthened to highlight the novelty of the method. Some of the components of the model are standard: Gaussian-inverse-Wishart model (equations 4, 5), choosing T=\nu I that leads to adjustment of eigenvalues (equations 10, 11). The authors should highlight main components of their method that leads to improved performance compared to existing methods.

We thank the Reviewer for this valuable feedback. We have updated the Methods, and added a section detailing the application to imputing GWAS summary statistics:

2 Methods

In Section 2.1 we introduce the explicit whitening transformations, and in Section 2.2 we reframe them from the perspective of a probabilistic model. We consider estimating a tuning parameter using an empirical Bayes approach in Section 2.3, and consider statistical properties and interpretation of the probabilistic whitening transformation in Section 2.4. In Section 2.5, we consider computational methods to apply the probabilistic whitening transform in high and ultra-high dimensional settings. In Section 2.6, we apply the transform to the problem of imputing regression summary statistics of ungenotyped variants.

2.6 Application to imputing z-statistics

Consider a matrix $X_{n\times p}$ of p genetic variants from n individuals, and a vector of continuous trait values y. Standard analysis in genome-wide association studies performs a linear regression to test association of each genetic variant with the trait. Ignoring covariates here for simplicity, this estimates a regression coefficient for the jth variant as $\hat{\beta}_j = (x_j^T x_j)^{-1} x_j^T y$, and produces a corresponding z-statistic, z_j. Within a local genomic region, genetic variants can be highly correlated due to linkage disequilibrium (LD). Therefore, even if the kth genetic variant is not directly observed, its z-statistic can be imputed from the set of observed z-statistics and the correlation structure between variants in the region. Following Pasaniuc, et al. [13], let $\Sigma_{p\times p}$ be the correlation matrix between all pairs of genetic variants, and partition the genetic variants into two sets with j storing indices with observed z-statistics and k storing indices without z-statistics. We would like to impute the values of z_k given observed values of z_j and Σ. If we model the z-statistics as a multivariate Gaussian

$$z \sim \mathcal{N}(0, \Sigma), \tag{20}$$

then the conditional distribution of z_k given z_j is

$$z_k|z_j \sim \mathcal{N}\left(\Sigma_{k,j}\Sigma_{j,j}^{-1}z_j,\, \Sigma_{j,j} - \Sigma_{k,j}\Sigma_{j,j}^{-1}\Sigma_{k,j}^T\right). \tag{21}$$

The imputed z-statistics is then

$$\hat{z}_k|z_j = \Sigma_{k,j}\Sigma_{j,j}^{-1}z_j. \tag{22}$$

This also works for logistic regression in case-control studies.

So far, we have assumed that the correlation matrix Σ is known. But in practice, this 'LD matrix' is estimated from a 'reference panel' of a modest number of individuals that are not included in the genome-wide association study. Therefore, inverting $\Sigma_{j,j}$ requires some regularization as p becomes large in comparison to n.

In the context of the current work, Equation (22) can be in interpreted as an out-of-sample whitening transformation of the observed z-statistics, z_j, and the cross-correlation matrix, $\Sigma_{k,j}$. Letting $\hat{\Sigma}_{\lambda,j,j}$ be

the regularized estimate of the correlation matrix, then the imputed z-statistic is

$$\left[\Sigma_{k,j}\hat{\Sigma}_{\lambda,j,j}^{-1/2}\right]\left[\hat{\Sigma}_{\lambda,j,j}^{-1/2}z_j\right]. \tag{23}$$

Applying this transformation by producing $\hat{\Sigma}_{\lambda,j,j}$ directly is cubic time in p_j, the number of genetic variants in set j. But applying the high dimensional whitening transformation of Equation (19) reduces this time substantially to $\mathcal{O}\left(n^2 p_j\right)$ when $n < p_j$. Imputation then depends on the observed z-statistics from the cross-correlation matrix, and the SVD of X.

This can be efficiently applied to real genotype data using **Algorithm 1** to impute summary statistics at indices k given summary statistics for variants at indices j, and genotype data from the reference panel at indices that are the union of k and j. The algorithm is then applied to every window in the genome.

Algorithm 1 Impute summary statistics with probabilistic whitening

```
function WHITEN(A, U_1, d⃗, V_1, ν, λ)          ▷ whiten matrix A given SVD and shrinkage parameters
  return (AV_1)diag[(1 − λ)d⃗^2 + λν]^(−1/2) V_1^T + λ^(−1/2) ν^(−1/2) (A − (YV_1)V_1^T)
end function

function IMPUTEZ(z_j, X_union(k,j))
   U_1, d⃗, V_1 ← svd(X_j)                       ▷ SVD of variants at indices j
   λ, ν ← EB(d⃗)                                 ▷ empirical Bayes estimate of shrinkage parameters
   Σ_k,j ← cor(X_k, X_j)                        ▷ compute cross-correlation matrix
   Σ̃_k,j ← WHITEN(Σ_k,j, U_1, d⃗, V_1, ν, λ)      ▷ whiten cross-correlation matrix
   z̃_j ← WHITEN(z_j, U_1, d⃗, V_1, ν, λ)          ▷ whiten vector of z-statistics
   ẑ_k ← Σ̃_k,j z̃_j                              ▷ imputed z-statistics for variants at indeces k
   return ẑ_k
end function
```

3. Figure 4 and 5: Improvement of GIW-EB compared to pre-chosen \lambda=0.1 seems to be minimal. It would be helpful to comment when the EB method leads to better performance and when it does not.

We have added the following text to clarify when the EB methods performs best.

Overall, the GIW-EB method consistently gives the lowest out-of-sample root mean square error across a range of p values in the four simulation scenarios (**Figure S1-4**). The low rank GIW-EB method is competitive in Scenarios 1 and 3, were the true covariance is low rank. GIW-EB shows the largest improvement compared to other methods when the correlation matrix is low rank, and the eigen-spectrum is dominated by the first few eigen-values.

We have added the following text in the Results based on the analysis of 1000 Genomes data

methods (**Figure 4B**). Error profiles and computational time was similar for individuals of African and Asian ancestry (**Figure S5A,B**), with GIW-EB giving lower error than fixing $\lambda = 0.01$ in 75.2% to 91.2% of genome windows, depending on the population (**Figure S5C**).

And added Figure S5C:

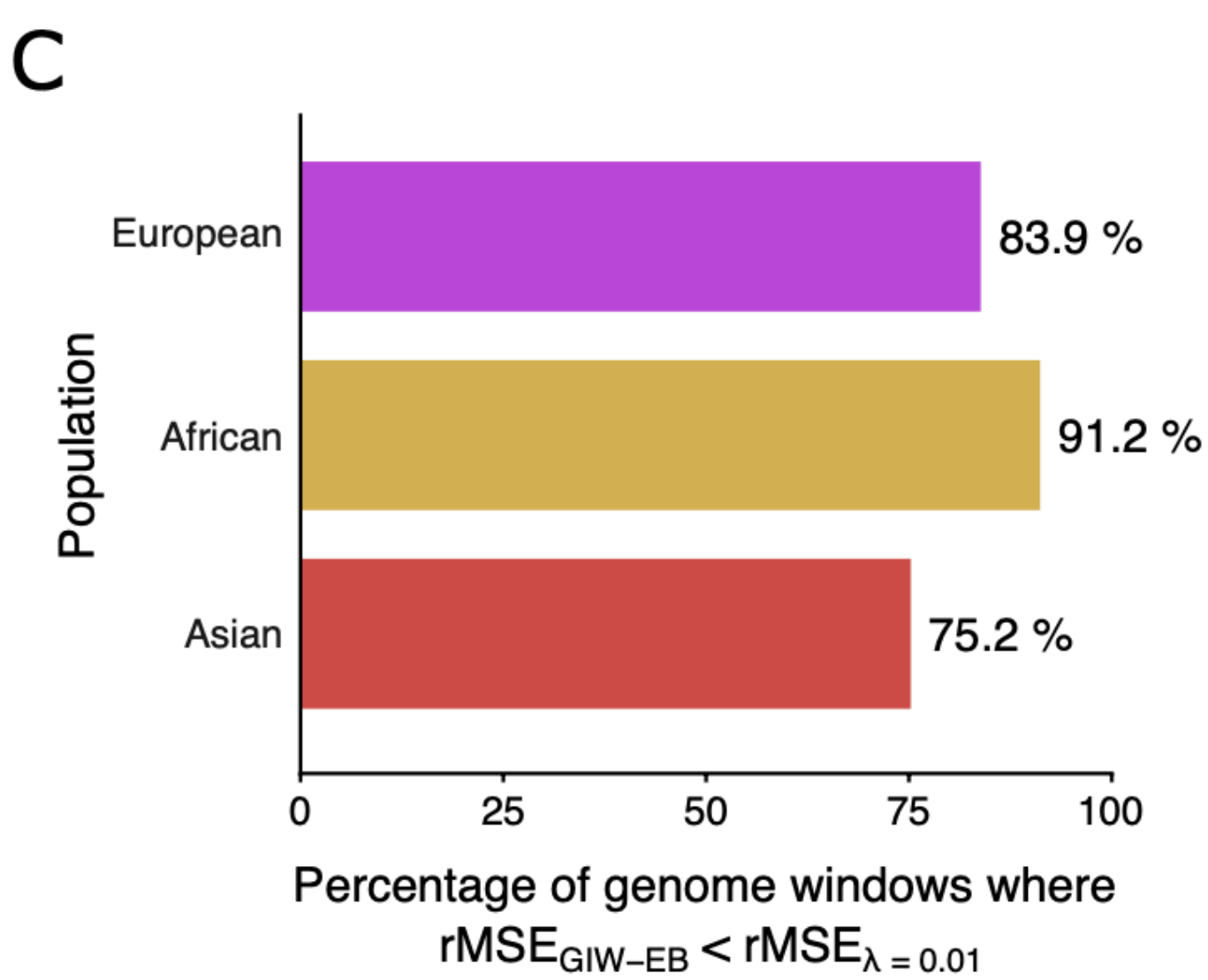

axes, the value is shown in white text. **B**) Run time of each method shown on a $\log_{10}$ scale. **C**) For each population, show the percentage of genome windows where the rMSE of GIW-EB is less than the rMSE from setting $\lambda = 0.01$.

4. Abstract: It is unclear what “First principles” means here. This term should be defined.

Agreed. We have rewritten the Abstract

5. Page 4: Should header “2 Results” be “2 Methods”?

Fixed.

6. Page 10 above equation 17: “whiting transform” should be “whitening transform”.

Fixed.

Reviewer #2:

The authors consider the task of whitening data for downstream analysis. They consider two challenges: 1) robustness in the high-dimensionsal regime (motivating shrinkage) and 2) scalability. They address 1 via a Bayesian formulation that enables empirical Bayesian selection of the regularization parameter lambda, and 2 via linear algebra manipulation of the SVD of the data. They present convincing results on simulated data, LD matrices, GWAS sum stat imputation and infering STR-eQTLs from SNP-eQTLs.

The manuscript is reasonably well written although I wonder if the flow could be improved. In several places they need to say "this will become relevant later". Possibly some reordering could improve clarity. I think a pseudoalgorithm might also help to show how all the pieces fit together - the lambda selection is particularly vague.

> We thank the Reviewer for this valuable feedback. We have improved text in the Abstract, Introduction and Methods.
>
> Statistical methods often make assumptions about independence between the samples or features of a dataset, yet correlation structure is ubiquitous in real data. In statistical genetics, correlation between regression summary statistics due to linkage disequilibrium (LD) between nearby variants complicates downstream analysis. Many methods use LD reference panels to account for this correlation. Whitening transformations comprise a family of methods to remove correlation structure, and are used implicitly by many common models. Here we propose a probabilistic model for data whitening, and propose parameter estimates and efficient algorithms to improve statistical and computational performance. In an application to impute z-statistics from unobserved genetic variants from a genome-wide association study of schizophrenia, the probabilistic whitening transformation, had the lowest mean square error while being up to an order of magnitude faster than other methods. Using this approach, we also identify tandem repeats that explain genetic regulatory signals for disease-relevant genes. Analyses are implemented in our novel open source R packages `decorrelate` and `imputez`.

1 Introduction

Correlation structure is ubiquitous in real world datasets. Yet statistical models often make assumptions about independence between a dataset's rows or columns that are not satisfied practice. Whitening transformations are a widely used preprocessing step to remove correlation structure in the data before performing other analyses. In general, for a data matrix $Y_{n \times p}$ with n samples and p features, the procedure computes the sample covariance matrix between features, $\Sigma_{p \times p}$, and transforms the observed data by post-multiplying by the non-unique whitening matrix $\Sigma^{-\frac{1}{2}}$ defined using standard linear algebra [1]. Transforming rows instead follows a complementary procedure.

Whitening can be used explicitly in preprocessing, but more often it is used implicitly when a covariance matrix is used internally to account for non-independence of samples or features and the whitening is implied by the linear algebra of the internal calculations. Indeed, many widely used methods such as multivariate regression, canonical correlation analysis, linear and quadratic discriminate analysis, Gaussian mixture models, Maholanobis distance, multivariate outlier detection, and linear mixed models can be reframed using an implicit whitening transformation [2–6].

The field of statistical genetics makes extensive use of implicit whitening transformations in order to account for the correlation between nearby genetic variants. This correlation structure is termed 'linkage disequlibirum' or 'LD' and is a major complication in downstream analysis of regression summary statistics (i.e. coefficient estimates or z-statistics) from genome-wide association studies [7]. It is common practice to estimate the correlation matrices from an external 'reference panel' of samples not included in the study of interest [7]. These 'out-of-sample' estimates are then used either as a preprocessing step to transform the summary statistics to be approximately uncorrelated, or internally in a statistical model. This approach has become standard in the field for phenotype prediction [8, 9], statistical fine-mapping [10–12], imputing z-statistics [13, 14], detecting anomalous z-statistics [15], multiple testing correction [16], identifying genome annotations enriched for risk variants [17], and simulating knockoff datasets with similar correlation structure [18]. While the literature on whitening transformations has focused on estimating and applying the transform on the same data [1], an understanding of the performance of these out-of-sample applications has been lacking [19].

Here, we examine the explicit whitening transformation and its properties in order to improve performance in a cases where it is used implicitly. This can improve out-of-sample performance when using modestly sized reference panels, and substantially reduce computational time.

2 Methods

In Section 2.1 we introduce the explicit whitening transformations, and in Section 2.2 we reframe them from the perspective of a probabilistic model. We consider estimating a tuning parameter using an empirical Bayes approach in Section 2.3, and consider statistical properties and interpretation of the probabilistic whitening transformation in Section 2.4. In Section 2.5, we consider computational methods to apply the probabilistic whitening transform in high and ultra-high dimensional settings. In Section 2.6, we apply the transform to the problem of imputing regression summary statistics of ungenotyped variants.

2.6 Application to imputing z-statistics

Consider a matrix $X_{n \times p}$ of p genetic variants from n individuals, and a vector of continuous trait values y. Standard analysis in genome-wide association studies performs a linear regression to test association of each genetic variant with the trait. Ignoring covariates here for simplicity, this estimates a regression coefficient for the jth variant as $\hat{\beta}_j = (x_j^T x_j)^{-1} x_j^T y$, and produces a corresponding z-statistic, z_j. Within a local genomic region, genetic variants can be highly correlated due to linkage disequilibrium (LD). Therefore, even if the kth genetic variant is not directly observed, its z-statistic can be imputed from the set of observed z-statistics and the correlation structure between variants in the region. Following Pasaniuc, et al. [13], let $\Sigma_{p \times p}$ be the correlation matrix between all pairs of genetic variants, and partition the genetic variants into two sets with j storing indices with observed z-statistics and k storing indices without z-statistics. We would like to impute the values of z_k given observed values of z_j and Σ. If we model the z-statistics as a multivariate Gaussian

$$z \sim \mathcal{N}(0, \Sigma), \tag{20}$$

then the conditional distribution of z_k given z_j is

$$z_k | z_j \sim \mathcal{N}\left(\Sigma_{k,j}\Sigma_{j,j}^{-1} z_j,\ \Sigma_{j,j} - \Sigma_{k,j}\Sigma_{j,j}^{-1}\Sigma_{k,j}^T\right). \tag{21}$$

The imputed z-statistics is then

$$\hat{z}_k | z_j = \Sigma_{k,j}\Sigma_{j,j}^{-1} z_j. \tag{22}$$

This also works for logistic regression in case-control studies.

So far, we have assumed that the correlation matrix Σ is known. But in practice, this 'LD matrix' is estimated from a 'reference panel' of a modest number of individuals that are not included in the genome-wide association study. Therefore, inverting $\Sigma_{j,j}$ requires some regularization as p becomes large in comparison to n.

In the context of the current work, Equation (22) can be in interpreted as an out-of-sample whitening transformation of the observed z-statistics, z_j, and the cross-correlation matrix, $\Sigma_{k,j}$. Letting $\hat{\Sigma}_{\lambda,j,j}$ be

the regularized estimate of the correlation matrix, then the imputed z-statistic is

$$\left[\Sigma_{k,j}\hat{\Sigma}_{\lambda,j,j}^{-1/2}\right]\left[\hat{\Sigma}_{\lambda,j,j}^{-1/2}z_j\right]. \tag{23}$$

Applying this transformation by producing $\hat{\Sigma}_{\lambda,j,j}$ directly is cubic time in p_j, the number of genetic variants in set j. But applying the high dimensional whitening transformation of Equation (19) reduces this time substantially to $\mathcal{O}(n^2 p_j)$ when $n < p_j$. Imputation then depends on the observed z-statistics from the cross-correlation matrix, and the SVD of X.

This can be efficiently applied to real genotype data using **Algorithm 1** to impute summary statistics at indices k given summary statistics for variants at indices j, and genotype data from the reference panel at indices that are the union of k and j. The algorithm is then applied to every window in the genome.

Algorithm 1 Impute summary statistics with probabilistic whitening

```
function WHITEN(A, U_1, d⃗, V_1, ν, λ)        ▷ whiten matrix A given SVD and shrinkage parameters
    return (A V_1) diag[(1 − λ) d⃗^2 + λν]^(−1/2) V_1^T + λ^(−1/2) ν^(−1/2) (A − (Y V_1) V_1^T)
end function

function IMPUTEZ(z_j, X_union(k,j))
    U_1, d⃗, V_1 ← svd(X_j)                       ▷ SVD of variants at indices j
    λ, ν ← EB(d⃗)                                  ▷ empirical Bayes estimate of shrinkage parameters
    Σ_{k,j} ← cor(X_k, X_j)                        ▷ compute cross-correlation matrix
    Σ̃_{k,j} ← WHITEN(Σ_{k,j}, U_1, d⃗, V_1, ν, λ)   ▷ whiten cross-correlation matrix
    z̃_j ← WHITEN(z_j, U_1, d⃗, V_1, ν, λ)           ▷ whiten vector of z-statistics
    ẑ_k ← Σ̃_{k,j} z̃_j                              ▷ imputed z-statistics for variants at indeces k
    return ẑ_k
end function
```

We agree that our description of estimating the lambda parameter in the main text Section 2.3 is limited. A more detailed description is provided in SI 5.4

Calling Theorem 1 a "theorem" is overselling imo: it's just an identity. Ditto for the Corollary.

Agreed. We now use “Identity”

This is useful methodology and the results are quite convincing. An additional valuable simulation experiment would be some way of showing the empirical Bayes lambda estimation is consisently good, not just "lucky" in the specific settings of the simulation. Possibly varying the SNR would achieve this.

We performed simulations under Scenarios 1-4

We consider simulations with data drawn from a multivariate Gaussian with $n = 500$ and the number of features p increasing from 20 to 2000, with correlation between features in each of 4 scenarios:

1) constant correlation of $\rho = 0.6$
2) auto-correlation with $\rho = 0.95$
3) 4 blocks of constant correlation of $\rho = 0.6$
4) 4 blocks of auto-correlation with $\rho = 0.95$

and results for a total of 1,200 conditions are shown in Figure 3, and Figures S1-4. We have added the following text to clarify when the EB methods performs best:

Overall, the GIW-EB method consistently gives the lowest out-of-sample root mean square error across a range of p values in the four simulation scenarios (**Figure S1-4**). The low rank GIW-EB method is competitive in Scenarios 1 and 3, were the true covariance is low rank. GIW-EB shows the largest improvement compared to other methods when the correlation matrix is low rank, and the eigen-spectrum is dominated by the first few eigen-values.

In addition we performed empirical evaluation using real genotype data from the 1000 Genomics Project in each of European, Asian and African super-populations (Figure 4, S5) in windows ranging from 100 to 11k genetic variants. An additional comparison between the GIW-EB method and fixing lambda = 0.01, shows that GIW-EB gives lower error across a large percentage of windows in the genome. We have added the following text in the Results

methods (**Figure 4B**). Error profiles and computational time was similar for individuals of African and Asian ancestry (**Figure S5A,B**), with GIW-EB giving lower error than fixing $\lambda = 0.01$ in 75.2% to 91.2% of genome windows, depending on the population (**Figure S5C**).

And added Figure S5C:

C

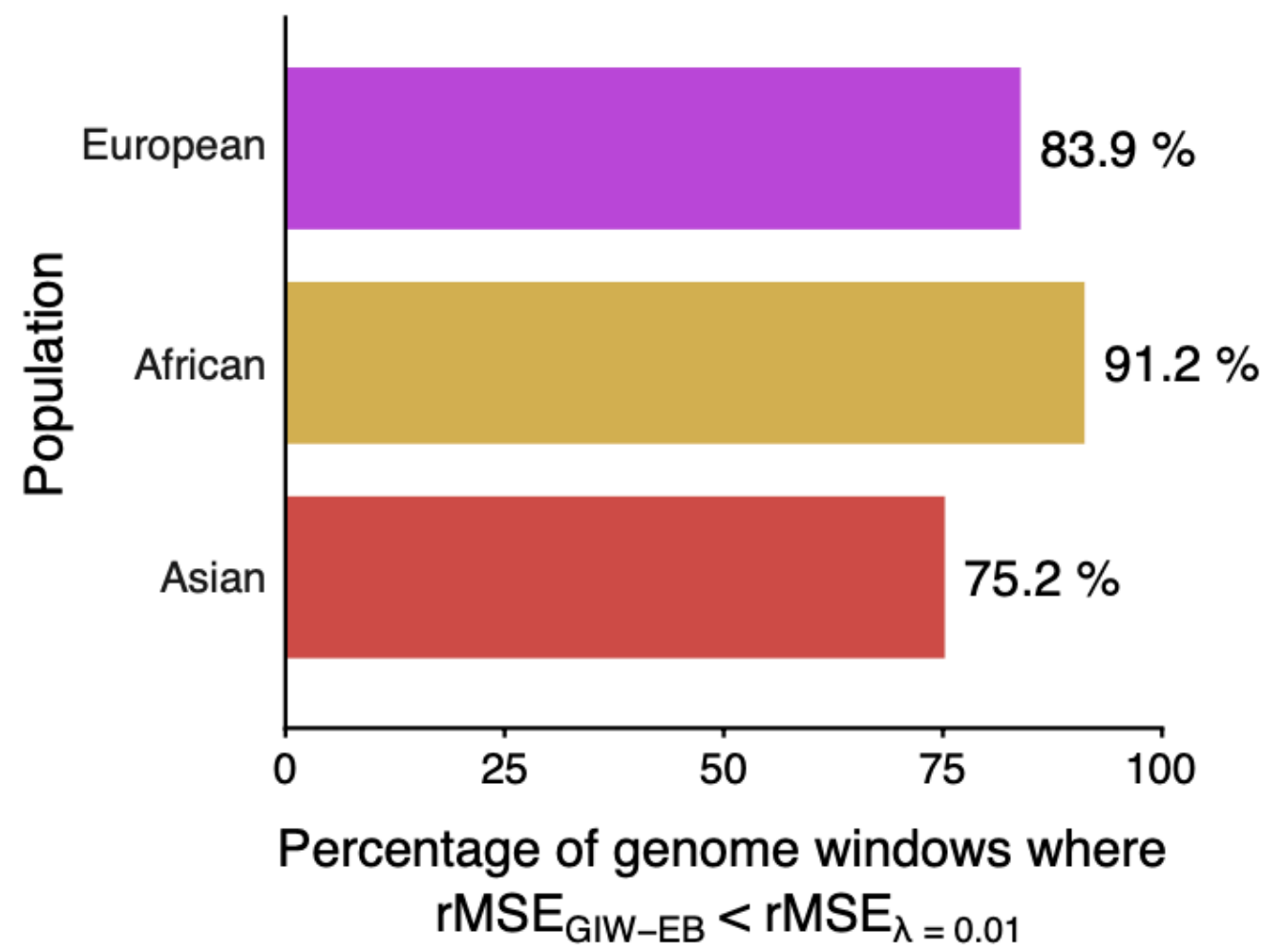

axes, the value is shown in white text. **B**) Run time of each method shown on a $\log_{10}$ scale. **C**) For each population, show the percentage of genome windows where the rMSE of GIW-EB is less than the rMSE from setting $\lambda = 0.01$.

Minor comments:

last sentence of first paragraph: "and" in wrong place

Fixed

The notation in eq 1 should be formally defined "[M,A,B] denotes a matrix random variable with E[Y]=M..." (i'm not sure it's actually valid how you have it with mu as a vector).

Agreed. We appreciate this feedback, and we have clarified the notation:

2.1 Introduction to whitening transformations

Consider observed data $Y_{n \times p}$ with n samples and p features drawn from a distribution with mean $M_{n \times p}$, identity covariance between rows, and covariance between features given by $\Sigma_{p \times p}$. Let

$$Y \sim [M, I_n, \Sigma] \tag{1}$$

indicate these properties without specifying particular distributional assumptions. Transforming the data by the non-unique whitening matrix $\Sigma^{-\frac{1}{2}}$ produces mean and covariances according to

$$Y\Sigma^{-\frac{1}{2}} \sim \left[M\Sigma^{-\frac{1}{2}}, I_n, I_p\right]. \tag{2}$$

—----------

posterior distribution of the covariance is also IW [41]. Formally,

$$Y|\Sigma \sim \mathcal{MN}(M, I_n, \Sigma) \tag{4}$$

$$\Sigma|T \sim \mathcal{IW}((\delta - p - 1)T, \delta) \tag{5}$$

—----------

5.2 Evaluating whitening performance

Let $\hat{A}$ be the whitening transformation learned from the observed data so that

$$Y\hat{A} \sim \left[M\hat{A}, I_n, \hat{A}^T\Sigma\hat{A}\right]. \tag{9}$$

end of p4, identify -> identity
eq4: should be Y|Sigma

after eq11: shrunk _towards nu_
p11: Due to its probabilistic formulation, _the_ whitening high-dimensional data

Fixed

Reviewer #3:

This is an interesting paper that proposes a new approach to whitening that scales to genome-scale data. However, I have some concerns about its appropriateness for publication in a genetics journal.

1. The paper claims that "the field of statistical genetics makes extensive use of whitening transformations in order to account for the correlation between nearby genetic variants". Could you give examples of these? I am familiar with many statgen methods that make use of correlation matrices, but I am not familiar with any that "transform summary statistics to be approximately uncorrelated".

> We thank the Reviewer for this valuable feedback. We rewritten the Abstract and Introduction to clarify that a whitening transformation is often *implicit* in widely used methods based on the linear algebra of the calculations. Here, we examine the explicit whitening transformation and its properties in order to improve performance in cases where it is used implicitly.
>
> Statistical methods often make assumptions about independence between the samples or features of a dataset, yet correlation structure is ubiquitous in real data. In statistical genetics, correlation between regression summary statistics due to linkage disequilibrium (LD) between nearby variants complicates downstream analysis. Many methods use LD reference panels to account for this correlation. Whitening transformations comprise a family of methods to remove correlation structure, and are used implicitly by many common models. Here we propose a probabilistic model for data whitening, and propose parameter estimates and efficient algorithms to improve statistical and computational performance. In an application to impute z-statistics from unobserved genetic variants from a genome-wide association study of schizophrenia, the probabilistic whitening transformation, had the lowest mean square error while being up to an order of magnitude faster than other methods. Using this approach, we also identify tandem repeats that explain genetic regulatory signals for disease-relevant genes. Analyses are implemented in our novel open source R packages `decorrelate` and `imputez`.

1 Introduction

Correlation structure is ubiquitous in real world datasets. Yet statistical models often make assumptions about independence between a dataset's rows or columns that are not satisfied practice. Whitening transformations are a widely used preprocessing step to remove correlation structure in the data before performing other analyses. In general, for a data matrix $Y_{n \times p}$ with n samples and p features, the procedure computes the sample covariance matrix between features, $\Sigma_{p \times p}$, and transforms the observed data by post-multiplying by the non-unique whitening matrix $\Sigma^{-\frac{1}{2}}$ defined using standard linear algebra [1]. Transforming rows instead follows a complementary procedure.

Whitening can be used explicitly in preprocessing, but more often it is used implicitly when a covariance matrix is used internally to account for non-independence of samples or features and the whitening is implied by the linear algebra of the internal calculations. Indeed, many widely used methods such as multivariate regression, canonical correlation analysis, linear and quadratic discriminate analysis, Gaussian mixture models, Maholanobis distance, multivariate outlier detection, and linear mixed models can be reframed using an implicit whitening transformation [2–6].

The field of statistical genetics makes extensive use of implicit whitening transformations in order to account for the correlation between nearby genetic variants. This correlation structure is termed 'linkage disequlibirum' or 'LD' and is a major complication in downstream analysis of regression summary statistics (i.e. coefficient estimates or z-statistics) from genome-wide association studies [7]. It is common practice to estimate the correlation matrices from an external 'reference panel' of samples not included in the study of interest [7]. These 'out-of-sample' estimates are then used either as a preprocessing step to transform the summary statistics to be approximately uncorrelated, or internally in a statistical model. This approach has become standard in the field for phenotype prediction [8, 9], statistical fine-mapping [10–12], imputing z-statistics [13, 14], detecting anomalous z-statistics [15], multiple testing correction [16], identifying genome annotations enriched for risk variants [17], and simulating knockoff datasets with similar correlation structure [18]. While the literature on whitening transformations has focused on estimating and applying the transform on the same data [1], an understanding of the performance of these out-of-sample applications has been lacking [19].

Here, we examine the explicit whitening transformation and its properties in order to improve performance in a cases where it is used implicitly. This can improve out-of-sample performance when using modestly sized reference panels, and substantially reduce computational time.

2. Thus, the use of whitening to impute z scores is, to me, novel in statgen. However, this novelty is not described beyond a reference to supplementary info. I think the application of whitening to imputation should be explained in more detail in the main paper.

We thank the Reviewer for this valuable feedback. We have added a new section to the Methods:

2.6 Application to imputing z-statistics

Consider a matrix $X_{n \times p}$ of p genetic variants from n individuals, and a vector of continuous trait values y. Standard analysis in genome-wide association studies performs a linear regression to test association of each genetic variant with the trait. Ignoring covariates here for simplicity, this estimates a regression coefficient for the jth variant as $\hat{\beta}_j = (x_j^T x_j)^{-1} x_j^T y$, and produces a corresponding z-statistic, z_j. Within a local genomic region, genetic variants can be highly correlated due to linkage disequilibrium (LD). Therefore, even if the kth genetic variant is not directly observed, its z-statistic can be imputed from the set of observed z-statistics and the correlation structure between variants in the region. Following Pasaniuc, et al. [13], let $\Sigma_{p \times p}$ be the correlation matrix between all pairs of genetic variants, and partition the genetic variants into two sets with j storing indices with observed z-statistics and k storing indices without z-statistics. We would like to impute the values of z_k given observed values of z_j and Σ. If we model the z-statistics as a multivariate Gaussian

$$z \sim \mathcal{N}(0, \Sigma), \tag{20}$$

then the conditional distribution of z_k given z_j is

$$z_k | z_j \sim \mathcal{N}\left(\Sigma_{k,j} \Sigma_{j,j}^{-1} z_j,\ \Sigma_{j,j} - \Sigma_{k,j} \Sigma_{j,j}^{-1} \Sigma_{k,j}^T\right). \tag{21}$$

The imputed z-statistics is then

$$\hat{z}_k | z_j = \Sigma_{k,j} \Sigma_{j,j}^{-1} z_j. \tag{22}$$

This also works for logistic regression in case-control studies.

So far, we have assumed that the correlation matrix Σ is known. But in practice, this 'LD matrix' is estimated from a 'reference panel' of a modest number of individuals that are not included in the genome-wide association study. Therefore, inverting $\Sigma_{j,j}$ requires some regularization as p becomes large in comparison to n.

In the context of the current work, Equation (22) can be in interpreted as an out-of-sample whitening transformation of the observed z-statistics, z_j, and the cross-correlation matrix, $\Sigma_{k,j}$. Letting $\hat{\Sigma}_{\lambda,j,j}$ be

the regularized estimate of the correlation matrix, then the imputed z-statistic is

$$\left[\Sigma_{k,j}\hat{\Sigma}_{\lambda,j,j}^{-1/2}\right]\left[\hat{\Sigma}_{\lambda,j,j}^{-1/2} z_j\right]. \tag{23}$$

Applying this transformation by producing $\hat{\Sigma}_{\lambda,j,j}$ directly is cubic time in p_j, the number of genetic variants in set j. But applying the high dimensional whitening transformation of Equation (19) reduces this time substantially to $\mathcal{O}\left(n^2 p_j\right)$ when $n < p_j$. Imputation then depends on the observed z-statistics from the cross-correlation matrix, and the SVD of X.

This can be efficiently applied to real genotype data using **Algorithm 1** to impute summary statistics at indices k given summary statistics for variants at indices j, and genotype data from the reference panel at indices that are the union of k and j. The algorithm is then applied to every window in the genome.

Algorithm 1 Impute summary statistics with probabilistic whitening

function WHITEN($A, U_1, \vec{d}, V_1, \nu, \lambda$) ▷ whiten matrix A given SVD and shrinkage parameters
  return $(AV_1)\mathrm{diag}\left[(1-\lambda)\vec{d}^2 + \lambda\nu\right]^{-\frac{1}{2}} V_1^T + \lambda^{-\frac{1}{2}}\nu^{-\frac{1}{2}}\left(A - (YV_1)V_1^T\right)$
end function

function IMPUTEZ(z_j, $X_{\mathrm{union}(k,j)}$)
  $U_1, \vec{d}, V_1 \leftarrow \mathrm{svd}(X_j)$ ▷ SVD of variants at indices j
  $\lambda, \nu \leftarrow \mathrm{EB}(\vec{d})$ ▷ empirical Bayes estimate of shrinkage parameters
  $\Sigma_{k,j} \leftarrow \mathrm{cor}(X_k, X_j)$ ▷ compute cross-correlation matrix
  $\tilde{\Sigma}_{k,j} \leftarrow$ WHITEN($\Sigma_{k,j}, U_1, \vec{d}, V_1, \nu, \lambda$) ▷ whiten cross-correlation matrix
  $\tilde{z}_j \leftarrow$ WHITEN($z_j, U_1, \vec{d}, V_1, \nu, \lambda$) ▷ whiten vector of z-statistics
  $\hat{z}_k \leftarrow \tilde{\Sigma}_{k,j}\tilde{z}_j$ ▷ imputed z-statistics for variants at indeces k
  return $\hat{z}_k$
end function

3. Relatedly, the use of the probabilistic model to calculate the covariance or precision matrix also appears novel. What other uses does this whitening approach have in statgen?

> In the new Introduction, we clarify how clarify a whitening transformation is often *implicit* in widely used methods based on the linear algebra of the calculations.

4. I am puzzled that you can model genotype data as multivariate normal, although equation (6) shows nicely that the wishart prior essentially regularises the MLE estimate. Could the use of multivariate normal for discrete data be justified?

> We agree that use of the multivariate normal distribution in the context of genotype data may be surprising. Of course, the genotype data are not drawn from a multivariate normal distribution. For example, many methods to impute genotypes adapt the Li and Stephens model (Genetics 2003, PMID: 14704198). In that context, the genotypes are random variables and the models compute the probability of observing some genotypes conditional on others. But those approaches are probabilisting models of the genotype data.
>
> Instead, our approach does not consider the distribution of the genotype data. It only considers a probabilistic model of the regression summary statistics conditional on the genotype data.

5. Here, the IW prior is centred around a scaled identity matrix. Could external data be used, eg if we have a large sample from a related population, and want to estimate Sigma in a smaller population?

> In principle, the method could use any prior matrix. However, the efficient whitening method we describe here only applies when the prior matrix is diagonal. We include this in the text now
>
> Setting the target matrix to be symmetric positive definite (SPD) ensures that the posterior expected covariance is also SPD [31]. Restricting T to be a minimally informative prior target corresponds to setting all prior covariances to zero. We focus here on the important case where the IW prior is centered around a scaled identity matrix, and we demonstrate that the estimate has exceptionally efficient computational scaling and produces estimates that are tractable to study analytically. Following previous work [23, 24, 31–33, 40] we set the target matrix to be $T = \nu I$ where $\nu = \text{tr}(\hat{\Sigma}_{MLE})/p$ is the average variance across all p features. This gives the important special case we consider here with
>
> $$\hat{\Sigma}_\lambda \;=\; (1-\lambda)\hat{\Sigma}_{MLE} + \lambda \nu I \tag{8}$$
>
> where $\hat{\Sigma}_\lambda$ is defined as the posterior expected covariance from the probabilistic model.

6. What is the OOS RMSE actually for in Fig 2 - is this for z scores or genotypes? Can you describe the simulations - what is being simulated, what is p in Fig 2B? The empirical OOS with varying lambda (black curve) is very smooth - how are you calculating this? What are these other methods for estimating lambda? What method should be used to estimate lambda in real world data, as none of these methods come close to the purple point?

> The simulation in Figure 2 was the same as in Scenario 3 and Figure 3. But based on feedback from all Reviewers, we have modified the Results section and removed this panel in order to improve flow and clarity.

7. In Fig 3, the simulated data are multivariate normal. What if they were scaled binomial, as genotype data are?

> We thank the Reviewer for this suggestion. Indeed, the simulations in Figure 3 use data drawn from a multivariate normal, while real genotype data is not. Instead of using simulated genotype to evaluate performance, we tested empirical performance of the 10 whitening methods on real data from the 1000 Genomes Project. Performance on European individuals is shown in Figure 4, and analysis of donors of African and Asian ancestry is shown in Figure S5.

8. In fig 5 you do not compare to the Pasaniuc method for z score imputation that you cite, ImpG, or any of the other existing statgen methods such as ssimp. Comparison to existing methods is required. Further, most applications would use a much larger reference panel. Does the whitening approach to this problem also benefit from larger reference panels?

> The ImpG method and SSimp method are very similar and both use the multivariate normal method that we apply here. The differences are just 1) the window size to select how many nearby variants to use from imputation and 2) how to select the lambda shrinkage value. At a practical level, we have not been able to download the ImpG software, as it is from 2014 and not available from its listed website. For SSimp, we were not able to change the window size or get it to work on the multiallelic tandem repeats that are the focus of this study.
>
> The question about the size of the reference panel is interesting. In this study we focus on imputing summary statistics for tandem repeats (TRs). Ziaei Jam (Nat Comm, 2023, PMID: 37872149) generated a reference map of 1.7 TRs from 3550 donors from the 1000 Genomes Project and H3Africa using long read sequencing, and we used a subset of 407 donors of European ancestry in our analysis. Of course, SNP

reference panels will have much larger sample size. The statistical and computational performance of summary statistic imputation will depend on 1) the size of the reference panel, 2) the number of nearby variants to include, and 3) the allele frequency of the target variant. The impact of these variables is important to consider in future work, but is outside the scope of the current study.
