# Supplementary material for: Fast probabilistic whitening transformation for ultra-high dimensional genetic data
Source: PLoS Genet. 2026 Oct 28;22(10):e1012320. doi: 10.1371/journal.pgen.1012320 (PMC13734586; doi:10.1371/journal.pgen.1012320)
Supplement: Peer review history

# Peer review: Decision Letter 0

**DOI:** 10.1371/journal.pgen.1012320.r001

**Authors:** Michael Epstein, Xiang Zhou

21 May 2026

PGENETICS-D-26-00375

Fast Probabilistic Whitening Transformation for Ultra-High Dimensional Genetic Data

PLOS Genetics

Dear Dr. Hoffman,

Thank you for submitting your manuscript to PLOS Genetics. After careful consideration, we feel that it has merit but does not fully meet PLOS Genetics's publication criteria as it currently stands. Therefore, we invite you to submit a revised version of the manuscript that addresses the points raised during the review process.

Please submit your revised manuscript by Jul 20 2026 11:59PM. If you will need more time than this to complete your revisions, please reply to this message or contact the journal office at plosgenetics@plos.org. When you're ready to submit your revision, log on to https://www.editorialmanager.com/pgenetics/ and select the 'Submissions Needing Revision' folder to locate your manuscript file.

Please include the following items when submitting your revised manuscript:

* A letter that responds to each point raised by the editor and reviewer(s). You should upload this letter as a separate file labeled 'Response to Reviewers'. This file does not need to include responses to any formatting updates and technical items listed in the 'Journal Requirements' section below.

* A marked-up copy of your manuscript that highlights changes made to the original version. You should upload this as a separate file labeled 'Revised Manuscript with Track Changes'.

* An unmarked version of your revised paper without tracked changes. You should upload this as a separate file labeled 'Manuscript'.

If you would like to make changes to your financial disclosure, competing interests statement, or data availability statement, please make these updates within the submission form at the time of resubmission. Guidelines for resubmitting your figure files are available below the reviewer comments at the end of this letter.

As the corresponding author, your ORCID iD is verified in the submission system and will appear in the published article. PLOS supports the use of ORCID, and we encourage all coauthors to register for an ORCID iD and use it as well. Please encourage your coauthors to verify their ORCID iD within the submission system before final acceptance, as unverified ORCID iDs will not appear in the published article. Only the individual author can complete the verification step; PLOS staff cannot verify ORCID iDs on behalf of authors.

We look forward to receiving your revised manuscript.

Kind regards,

Xiang Zhou, Ph.D.

Academic Editor

PLOS Genetics

Michael P. Epstein

Section Editor

PLOS Genetics

Aimée Dudley

Editor-in-Chief

PLOS Genetics

Anne Goriely

Editor-in-Chief

PLOS Genetics

**Journal Requirements:**

If the reviewer comments include a recommendation to cite specific previously published works, please review and evaluate these publications to determine whether they are relevant and should be cited. There is no requirement to cite these works unless the editor has indicated otherwise.

1) Please ensure that the CRediT author contributions listed for every co-author are completed accurately and in full.

At this stage, the following Authors/Authors require contributions: Gabriel Hoffman, Christian Dillard, Kiran Girdhar, and Panos Roussos. Please ensure that the full contributions of each author are acknowledged in the "Add/Edit/Remove Authors" section of our submission form.

The list of CRediT author contributions may be found here: https://journals.plos.org/plosgenetics/s/authorship#loc-author-contributions

2) We ask that a manuscript source file is provided at Revision. Please upload your manuscript file as a .doc, .docx, .rtf or .tex. If you are providing a .tex file, please upload it under the item type u2018LaTeX Source Fileu2019 and leave your .pdf version as the item type u2018Manuscriptu2019.

3) Please provide an Author Summary. This should appear in your manuscript between the Abstract (if applicable) and the Introduction, and should be 150-200 words long. The aim should be to make your findings accessible to a wide audience that includes both scientists and non-scientists. Sample summaries can be found on our website under Submission Guidelines:

https://journals.plos.org/plosgenetics/s/submission-guidelines#loc-parts-of-a-submission

4) Your manuscript is missing the following sections: Abstract, and Methods.  Please ensure all required sections are present and in the correct order. Make sure section heading levels are clearly indicated in the manuscript text, and limit sub-sections to 3 heading levels. An outline of the required sections can be consulted in our submission guidelines here:

https://journals.plos.org/plosgenetics/s/submission-guidelines#loc-parts-of-a-submission

5) Please upload all main figures as separate Figure files in .tif or .eps format. For more information about how to convert and format your figure files please see our guidelines:

https://journals.plos.org/plosgenetics/s/figures

6) We notice that your supplementary Figures, and Tables are included in the manuscript file. Please remove them and upload them with the file type 'Supporting Information'. Please ensure that each Supporting Information file has a legend listed in the manuscript after the references list.

7) We note that your Data Availability Statement is currently as follows: "All data is publicly available in the original publications.". Please confirm at this time whether or not your submission contains all raw data required to replicate the results of your study. Authors must share the “minimal data set” for their submission. PLOS defines the minimal data set to consist of the data required to replicate all study findings reported in the article, as well as related metadata and methods (https://journals.plos.org/plosone/s/data-availability#loc-minimal-data-set-definition).

For example, authors should submit the following data:

1) The values behind the means, standard deviations and other measures reported;

2) The values used to build graphs;

3) The points extracted from images for analysis..

Authors do not need to submit their entire data set if only a portion of the data was used in the reported study.

If your submission does not contain these data, please either upload them as Supporting Information files or deposit them to a stable, public repository and provide us with the relevant URLs, DOIs, or accession numbers. For a list of recommended repositories, please see https://journals.plos.org/plosone/s/recommended-repositories.

If there are ethical or legal restrictions on sharing a de-identified data set, please explain them in detail (e.g., data contain potentially sensitive information, data are owned by a third-party organization, etc.) and who has imposed them (e.g., an ethics committee). Please also provide contact information for a data access committee, ethics committee, or other institutional body to which data requests may be sent. If data are owned by a third party, please indicate how others may request data access.

8) Please amend your detailed Financial Disclosure statement. This is published with the article. It must therefore be completed in full sentences and contain the exact wording you wish to be published.

1) State what role the funders took in the study. If the funders had no role in your study, please state: "The funders had no role in study design, data collection and analysis, decision to publish, or preparation of the manuscript."

2) If any authors received a salary from any of your funders, please state which authors and which funders..

If you did not receive any funding for this study, please simply state: u201cThe authors received no specific funding for this work.u201d

**Reviewers' comments:**

Reviewer's Responses to Questions

**Comments to the Authors:**

**Please note here if the review is uploaded as an attachment.**

Reviewer #1: Whitening is widely used in many analyses of genetic data. This manuscript proposes a new method for performing whitening transformations accurately and efficiently. Overall, the paper addresses an important problem, and the proposed method has the potential for broad applications in genetics and beyond. I have the following comments.

1. The abstract and introduction are more appropriate for a statistical audience. The description or scientific motivations is brief. For example, why is probabilistic whitening important for a broad audience in the field of genetics? Although the authors mentioned phenotype prediction, statistical fine-mapping, imputing z-statistics, detecting anomalous z-statistics, multiple testing correction, etc, in the introduction, they did not elaborate on the limitation of current whitening methods in the context of these analyses and how they can influence downstream findings. Discussing these motivations early in the Introduction could appeal to more researchers who may be interested in using the method. Similarly, starting the abstract with a bit more scientific motivation would help.

2. The methods should be strengthened to highlight the novelty of the method. Some of the components of the model are standard: Gaussian-inverse-Wishart model (equations 4, 5), choosing T=\nu I that leads to adjustment of eigenvalues (equations 10, 11). The authors should highlight main components of their method that leads to improved performance compared to existing methods.

3. Figure 4 and 5: Improvement of GIW-EB compared to pre-chosen \lambda=0.1 seems to be minimal. It would be helpful to comment when the EB method leads to better performance and when it does not.

4. Abstract: It is unclear what “First principles” means here. This term should be defined.

5. Page 4: Should header “2 Results” be “2 Methods”?

6. Page 10 above equation 17: “whiting transform” should be “whitening transform”.

Reviewer #2: The authors consider the task of whitening data for downstream analysis. They consider two challenges: 1) robustness in the high-dimensionsal regime (motivating shrinkage) and 2) scalability. They address 1 via a Bayesian formulation that enables empirical Bayesian selection of the regularization parameter lambda, and 2 via linear algebra manipulation of the SVD of the data. They present convincing results on simulated data, LD matrices, GWAS sum stat imputation and infering STR-eQTLs from SNP-eQTLs.

The manuscript is reasonably well written although I wonder if the flow could be improved. In several places they need to say "this will become relevant later". Possibly some reordering could improve clarity. I think a pseudoalgorithm might also help to show how all the pieces fit together - the lambda selection is particularly vague.

Calling Theorem 1 a "theorem" is overselling imo: it's just an identity. Ditto for the Corollary.

This is useful methodology and the results are quite convincing. An additional valuable simulation experiment would be some way of showing the empirical Bayes lambda estimation is consisently good, not just "lucky" in the specific settings of the simulation. Possibly varying the SNR would achieve this.

Minor comments:

last sentence of first paragraph: "and" in wrong place

The notation in eq 1 should be formally defined "[M,A,B] denotes a matrix random variable with E[Y]=M..." (i'm not sure it's actually valid how you have it with mu as a vector)

end of p4, identify -> identity

eq4: should be Y|Sigma

after eq11: shrunk _towards nu_

p11: Due to its probabilistic formulation, _the_ whitening high-dimensional data

Reviewer #3: This is an interesting paper that proposes a new approach to whitening that scales to genome-scale data. However, I have some concerns about its appropriateness for publication in a genetics journal.

1. The paper claims that "the field of statistical genetics makes extensive use of whitening transformations in order to account for the correlation between nearby genetic variants". Could you give examples of these? I am familiar with many statgen methods that make use of correlation matrices, but I am not familiar with any that "transform summary statistics to be approximately uncorrelated".

2. Thus, the use of whitening to impute z scores is, to me, novel in statgen. However, this novelty is not described beyond a reference to supplementary info. I think the application of whitening to imputation should be explained in more detail in the main paper.

3. Relatedly, the use of the probabilistic model to calculate the covariance or precision matrix also appears novel. What other uses does this whitening approach have in statgen?

4. I am puzzled that you can model genotype data as multivariate normal, although equation (6) shows nicely that the wishart prior essentially regularises the MLE estimate. Could the use of multivariate normal for discrete data be justified?

5. Here, the IW prior is centred around a scaled identity matrix. Could external data be used, eg if we have a large sample from a related population, and want to estimate Sigma in a smaller population?

6. What is the OOS RMSE actually for in Fig 2 - is this for z scores or genotypes? Can you describe the simulations - what is being simulated, what is p in Fig 2B? The empirical OOS with varying lambda (black curve) is very smooth - how are you calculating this? What are these other methods for estimating lambda? What method should be used to estimate lambda in real world data, as none of these methods come close to the purple point?

7. In Fig 3, the simulated data are multivariate normal. What if they were scaled binomial, as genotype data are?

8. In fig 5 you do not compare to the Pasaniuc method for z score imputation that you cite, ImpG, or any of the other existing statgen methods such as ssimp. Comparison to existing methods is required. Further, most applications would use a much larger reference panel. Does the whitening approach to this problem also benefit from larger reference panels?

**********

**Have all data underlying the figures and results presented in the manuscript been provided?**

Large-scale datasets should be made available via a public repository as described in the *PLOS Genetics*  data availability policy, and numerical data that underlies graphs or summary statistics should be provided in spreadsheet form as supporting information.

Reviewer #1: None

Reviewer #2: Yes

Reviewer #3: Yes

**********

PLOS authors have the option to publish the peer review history of their article (what does this mean?). If published, this will include your full peer review and any attached files.

If you choose “no”, your identity will remain anonymous but your review may still be made public.

**Do you want your identity to be public for this peer review?**  For information about this choice, including consent withdrawal, please see our Privacy Policy.

Reviewer #1: No

Reviewer #2: No

Reviewer #3: No

**Figure resubmission:**

While revising your submission, we strongly recommend that you use PLOS’s NAAS tool (https://ngplosjournals.pagemajik.ai/artanalysis) to test your figure files. NAAS can convert your figure files to the TIFF file type and meet basic requirements (such as print size, resolution), or provide you with a report on issues that do not meet our requirements and that NAAS cannot fix.

After uploading your figures to PLOS’s NAAS tool - https://ngplosjournals.pagemajik.ai/artanalysis, NAAS will process the files provided and display the results in the "Uploaded Files" section of the page as the processing is complete. If the uploaded figures meet our requirements (or NAAS is able to fix the files to meet our requirements), the figure will be marked as "fixed" above. If NAAS is unable to fix the files, a red "failed" label will appear above. When NAAS has confirmed that the figure files meet our requirements, please download the file via the download option, and include these NAAS processed figure files when submitting your revised manuscript.

**Reproducibility:**

To enhance the reproducibility of your results, we recommend that authors of applicable studies deposit laboratory protocols in protocols.io, where a protocol can be assigned its own identifier (DOI) such that it can be cited independently in the future. Additionally, PLOS ONE offers an option to publish peer-reviewed clinical study protocols. Read more information on sharing protocols at https://plos.org/protocols?utm_medium=editorial-email&utm_source=authorletters&utm_campaign=protocols

# Author comment: Author response to Decision Letter 1

**DOI:** 10.1371/journal.pgen.1012320.r002

13 Aug 2026

Attachment Submitted filename: ImputeZ response.pdf

# Peer review: Decision Letter 1

**DOI:** 10.1371/journal.pgen.1012320.r003

**Authors:** Michael Epstein, Xiang Zhou

17 Sep 2026

Dear Dr Hoffman,

We are pleased to inform you that your manuscript entitled "Fast Probabilistic Whitening Transformation for Ultra-High Dimensional Genetic Data" has been editorially accepted for publication in PLOS Genetics. Congratulations!

Before your submission can be formally accepted and sent to production you will need to complete our formatting changes, which you will receive in a follow up email. Please be aware that it may take several days for you to receive this email; during this time no action is required by you. Please note: the accept date on your published article will reflect the date of this provisional acceptance, but your manuscript will not be scheduled for publication until the required changes have been made.

Once your paper is formally accepted, an uncorrected proof of your manuscript will be published online ahead of the final version, unless you’ve already opted out via the online submission form. If, for any reason, you do not want an earlier version of your manuscript published online or are unsure if you have already indicated as such, please let the journal staff know immediately at plosgenetics@plos.org.

In the meantime, please log into Editorial Manager at https://www.editorialmanager.com/pgenetics/, click the "Update My Information" link at the top of the page, and update your user information to ensure an efficient production and billing process. Note that PLOS requires an ORCID iD for all corresponding authors. Therefore, please ensure that you have an ORCID iD and that it is validated in Editorial Manager. To do this, go to ‘Update my Information’ (in the upper left-hand corner of the main menu), and click on the Fetch/Validate link next to the ORCID field.  This will take you to the ORCID site and allow you to create a new iD or authenticate a pre-existing iD in Editorial Manager.

If you have a press-related query, or would like to know about making your underlying data available (as you will be aware, this is required for publication), please see the end of this email. If your institution or institutions have a press office, please notify them about your upcoming article at this point, to enable them to help maximise its impact. Inform journal staff as soon as possible if you are preparing a press release for your article and need a publication date.

Thank you again for supporting open-access publishing; we are looking forward to publishing your work in PLOS Genetics!

Yours sincerely,

Xiang Zhou, Ph.D.

Academic Editor

PLOS Genetics

Michael Epstein

Section Editor

PLOS Genetics

Aimée Dudley

Editor-in-Chief

PLOS Genetics

Anne Goriely

Editor-in-Chief

PLOS Genetics

www.plosgenetics.org

BlueSky: @plos.bsky.social

----------------------------------------------------

Comments from the reviewers (if applicable):

Reviewer's Responses to Questions

**Comments to the Authors:**

**Please note here if the review is uploaded as an attachment.**

Reviewer #1: The authors have addressed my questions.

Reviewer #2: The authors have addressed my concerns, I recommend acceptance.

**********

**Have all data underlying the figures and results presented in the manuscript been provided?**

Large-scale datasets should be made available via a public repository as described in the *PLOS Genetics*  data availability policy, and numerical data that underlies graphs or summary statistics should be provided in spreadsheet form as supporting information.

Reviewer #1: None

Reviewer #2: Yes

**********

PLOS authors have the option to publish the peer review history of their article (what does this mean?). If published, this will include your full peer review and any attached files.

If you choose “no”, your identity will remain anonymous but your review may still be made public.

**Do you want your identity to be public for this peer review?**  For information about this choice, including consent withdrawal, please see our Privacy Policy.

Reviewer #1: No

Reviewer #2: No

----------------------------------------------------

**Data Deposition**

If you have submitted a Research Article or Front Matter that has associated data that are not suitable for deposition in a subject-specific public repository (such as GenBank or ArrayExpress), one way to make that data available is to deposit it in the Dryad Digital Repository. As you may recall, we ask all authors to agree to make data available; this is one way to achieve that. A full list of recommended repositories can be found on our website.

The following link will take you to the Dryad record for your article, so you won't have to re‐enter its bibliographic information, and can upload your files directly:

http://datadryad.org/submit?journalID=pgenetics&manu=PGENETICS-D-26-00375R1

More information about depositing data in Dryad is available at http://www.datadryad.org/depositing. If you experience any difficulties in submitting your data, please contact help@datadryad.org for support.

Additionally, please be aware that our data availability policy requires that all numerical data underlying display items are included with the submission, and you will need to provide this before we can formally accept your manuscript, if not already present.

----------------------------------------------------

**Press Queries**

If you or your institution will be preparing press materials for this manuscript, or if you need to know your paper's publication date for media purposes, please inform the journal staff as soon as possible so that your submission can be scheduled accordingly. Your manuscript will remain under a strict press embargo until the publication date and time. This means an early version of your manuscript will not be published ahead of your final version. PLOS Genetics may also choose to issue a press release for your article. If there's anything the journal should know or you'd like more information, please get in touch via plosgenetics@plos.org.

# Editor report: Acceptance letter

**DOI:** 10.1371/journal.pgen.1012320.r004

**Authors:** Michael Epstein, Xiang Zhou

PGENETICS-D-26-00375R1

Fast Probabilistic Whitening Transformation for Ultra-High Dimensional Genetic Data

Dear Dr Hoffman,

We are pleased to inform you that your manuscript entitled "Fast Probabilistic Whitening Transformation for Ultra-High Dimensional Genetic Data" has been formally accepted for publication in PLOS Genetics! Your manuscript is now with our production department and you will be notified of the publication date in due course.

The corresponding author will soon be receiving a typeset proof for review, to ensure errors have not been introduced during production. Please review the PDF proof of your manuscript carefully, as this is the last chance to correct any errors. Please note that major changes, or those which affect the scientific understanding of the work, will likely cause delays to the publication date of your manuscript.

Soon after your final files are uploaded, unless you have opted out or your manuscript is a front-matter piece, the early version of your manuscript will be published online. The date of the early version will be your article's publication date. The final article will be published to the same URL, and all versions of the paper will be accessible to readers.

For Research Articles, you will receive an invoice from PLOS for your publication fee after your manuscript has reached the completed accept phase. If you receive an email requesting payment before acceptance or for any other service, this may be a phishing scheme. Learn how to identify phishing emails and protect your accounts at https://explore.plos.org/phishing.

Thank you again for supporting PLOS Genetics and open-access publishing. We are looking forward to publishing your work!

With kind regards,

Sharmila Kamatchi

PLOS Genetics

On behalf of:

The PLOS Genetics Team

Carlyle House, Carlyle Road, Cambridge CB4 3DN | United Kingdom

plosgenetics@plos.org | +44 (0) 1223-442823

plosgenetics.org | Twitter: @PLOSGenetics
